# Neurofuzzy logic predicts a fine-tuning metabolic reprogramming on elicited *Bryophyllum* PCSCs guided by salicylic acid

**DOI:** 10.3389/fpls.2022.991557

**Published:** 2022-09-23

**Authors:** Pascual García-Pérez, Eva Lozano-Milo, Leilei Zhang, Begoña Miras-Moreno, Mariana Landin, Luigi Lucini, Pedro P. Gallego

**Affiliations:** ^1^ Agrobiotech for Health, Plant Biology and Soil Science Department, Faculty of Biology, University of Vigo, Vigo, Spain; ^2^ Sustainable Food Process Department, Università Cattolica del Sacro Cuore, Piacenza, Italy; ^3^ Cluster de Investigación y Transferencia Agroalimentaria del Campus da Auga (CITACA), University of Vigo, Orense Campus, Ourense, Spain; ^4^ Pharmacology, Pharmacy, and Pharmaceutical Technology Department, I+D Farma (GI-1645), Faculty of Pharmacy, Instituto de Materiales de la Universidade de Santiago de Compostela (iMATUS) and Health Research Institute of Santiago de Compostela (IDIS), Universidade de Santiago de Compostela, Santiago de Compostela, Spain

**Keywords:** *Kalanchoe*, polyphenols, metabolic fingerprint, Untargeted metabolic profiling, plant biotechnology, medicinal plants

## Abstract

Novel approaches to the characterization of medicinal plants as biofactories have lately increased in the field of biotechnology. In this work, a multifaceted approach based on plant tissue culture, metabolomics, and machine learning was applied to decipher and further characterize the biosynthesis of phenolic compounds by eliciting cell suspension cultures from medicinal plants belonging to the *Bryophyllum* subgenus. The application of untargeted metabolomics provided a total of 460 phenolic compounds. The biosynthesis of 164 of them was significantly modulated by elicitation. The application of neurofuzzy logic as a machine learning tool allowed for deciphering the critical factors involved in the response to elicitation, predicting their influence and interactions on plant cell growth and the biosynthesis of several polyphenols subfamilies. The results indicate that salicylic acid plays a definitive genotype-dependent role in the elicitation of *Bryophyllum* cell cultures, while methyl jasmonate was revealed as a secondary factor. The knowledge provided by this approach opens a wide perspective on the research of medicinal plants and facilitates their biotechnological exploitation as biofactories in the food, cosmetic and pharmaceutical fields.

## Introduction

Elicitation of plant cell suspension cultures (PCSCs) constitutes a well-established and efficient strategy, largely used in the field of plant biotechnology, to enhance the accumulation of secondary metabolites, with a wide range of applications in different sectors, including cosmetical, pharmaceutical, food, and agricultural industries, among others. This phenomenon is based on the induction of eustress through an external factor or set of factors that promote a controlled stimulation of plant responses related to stress, thus reprogramming the pathways of cell signalling and favouring the biosynthesis of secondary metabolites. (Aguirre-Becerra et al., 2021). Controlled induction of plant secondary metabolism requires a reliable and stable plant system. *In vivo* systems under elicitation provide rare and contradictory results, which is why the establishment of the plant *in vitro* culture of the plant has traditionally been used (Kandoudi et al., 2022). The use of elicited PCSCs represents an advanced approach to producing secondary metabolites on a large scale. This technology allows the rapid growth of cells in axenic conditions, turning bioreactors into true biofactories with easy scaling up and great application prospects (Krasteva et al., 2021).

Two plant hormones have been exploited as elicitors in the field of biotechnology for the enhancement of secondary metabolism in PCSCs: methyl jasmonate (MJ) and salicylic acid (SA), which represent key molecules involved in the regulation of plant growth and stress tolerance. MJ is the master regulator of the general response to biotic stress in plants, mainly induced by microbiological infections, necrotic pathogens, and herbivore attacks (Nabi et al., 2021). SA presents a universal role in both abiotic and biotic stress tolerance, although its study has been mainly focused on its role in abiotic stress, towards different environmental threats, such as salinity, drought, cold, or UV radiation (Nandy et al., 2021). The exogenous application of both on PCSCs has had controversial results. The crosstalk between both molecules is not well understood to date, showing for instance a negative outcome in heavy metal-induced oxidative stress (Pandey et al., 2021), whereas they positively coordinate to induce tolerance against cold stress (Hossain et al., 2021). The effect of the combination of both elicitors on the secondary metabolism of plants is not yet fully defined. Further efforts are required to clarify this complex process. Novel approaches are needed to efficiently characterize and predict the effectiveness of MJ and SA in enhancing plant secondary metabolism.

Machine learning includes a powerful set of computational tools to efficiently unmask concealed patterns and interactions between variables, thus conferring a deep understanding of complex and multifactorial systems, as is the case with elicitation (Greener et al., 2022). In particular, the combination of artificial neural networks (ANNs) with fuzzy logic to give rise to neurofuzzy logic (NFL), has been successfully applied to understand and further characterize a plethora of different plant physiological processes (Gago et al., 2011; Nezami-Alanagh et al., 2018), from medicinal plant nutrition (García-Pérez et al., 2020b; Maleki et al., 2021; Arteta et al., 2022) to *in vitro* organogenesis (García-Pérez et al., 2020d). Moreover, to unravel the wide effect of elicitation on PCSCs, the application of metabolomics involves a multidimensional analytical technology to detect the metabolic fingerprinting associated with different physiological processes, such as the metabolic reprogramming induced by stress through untargeted metabolomics (UM) approaches (Li and Gaquerel, 2021).

The robustness and plasticity of both NFL and UM technologies allow the performance of a combinatorial approach to decipher the key factors affecting the biosynthesis of phenolic compounds on elicited medicinal PCSCs (García-Pérez and Gallego, 2022). In this work, NFL and UM were combined to that aim using three different species from the *Bryophyllum* subgenus, largely applied in the traditional medicine of Africa, South America, and Asia (García-Pérez et al., 2020a). In this sense, different *Bryophyllum*-derived formulations have been largely described as efficient treatment of a plethora of chronic diseases, as is the case of microbial infections, diabetes, respiratory and neurological diseases, inflammatory disorders, and cancer (Lozano-Milo et al., 2020). Such a pharmacological potential has been mostly attributed to the biosynthesis of two families of secondary metabolites: bufadienolides and phenolic compounds, the latter receiving little attention in the field of phytochemical research (García-Pérez et al., 2020a). As unexplored medicinal plants, the combination of UM with ML will provide a source of knowledge regarding the phytochemical potential associated with *Bryophyllum* species, enabling their large-scale application in different economically important sectors as biofactories of health-enhancing compounds. Indeed, the industrial potential of these medicinal plants has prompted the application of ML tools to decipher and predict key physiological processes *in vitro*, such as mineral nutrition (García-Pérez et al., 2020b), organogenesis (García-Pérez et al., 2020d), and the production of phenolic compounds by plant tissues (García-Pérez et al., 2020c; García-Pérez et al., 2021c) and PCSCs elicited with cyclodextrins (García-Pérez et al., 2019). Due to the extraordinary and multifaceted secondary metabolism attributed to *Bryophyllum* sp., these plants have been recently highlighted as paramount medicinal plants, which deserve biotechnological standardization thanks to their associated bioactivities, exhibiting a diversified antioxidant activity, cytotoxicity against a wide range of human cancer cell lines, and an extraordinary antimicrobial and antifungal activity against diverse pathogens (García-Pérez et al., 2021a; García-Pérez et al., 2021b). This work aims at conferring a novel multidisciplinary approach combining high throughput technology, such as UM, and machine learning tools like NFL, to provide insight into the production of phenolic compounds, by predicting the underlying interactions of universal elicitors, i.e.: MJ and SA on *Bryophyllum* PCSCs.

## Materials and methods

### Chemicals and reagents

All reagents for plant tissue and cell culture elicitation development were of the highest purity and purchased from Sigma Aldrich. All chemicals used for sample extraction and metabolomics analysis were LC-MS grade, supplied by VWR Chemicals (Milan, Italy).

### Plant cell suspension culture establishment

Epiphyllous plantlets from three *Bryophyllum* species, namely *Bryophyllum daigremontianum* Raym.-Hamet et Perr. (BD, syn. *Kalanchoe daigremontiana*), *Bryophyllum* × *houghtonii* D.B. Ward (BH, syn. *Kalanchoe daigremontiana* × *tubiflora*), and *Bryophyllum tubiflorum* Harv. (BT, syn. *Kalanchoe tubiflora*), were harvested from a local greenhouse, surface disinfected, and micropropagated in Murashige and Skoog medium (MS) (Murashige and Skoog, 1962) supplemented with 3% sucrose and 0.8% agar at pH = 5.8, as reported earlier (García-Pérez et al., 2020a). Leaves from 12-week-old *in vitro* cultured plants were employed for callus induction, using different culture media formulations for each species: MS medium with 0.5 mg L^-1^ 2,4-dichlorophenoxyacetic acid (2,4-D) and 1 mg L^-1^ 6-benzylaminopurine (BAP) for BD, half-macronutrient MS medium with 0.5 mg L^-1^ 2,4-D and 1 mg L^-1^ BAP for BH, and MS medium with 1 mg L^-1^ 2,4-D and 0.5 mg L^-1^ BAP for BT (García-Pérez et al., 2021b). All media were supplemented with 3% sucrose and 0.8% agar at pH = 5.8 (García-Pérez et al., 2019). Leaf segments (≅1 cm^2^) were then cultured in Petri dishes with solidified medium and incubated at 25°C in the dark for 4 weeks to promote callus formation. Afterward, calli were subcultured every 21 days under the same conditions. For the establishment of *Bryophyllum* cell suspension cultures, 21-day-old calli were transferred to the same media without agar at a cell density of 100 g per litre in 250-mL Erlenmeyer flasks. Cultures were then incubated at 25°C in the dark at 120 rpm in an orbital shaker and subcultured every 9 days by the half-dilution method.

### Elicitation experiments

Cell cultures from the third subculture were subjected to subsequent elicitation experiments with two elicitors, methyl jasmonate (MJ) and salicylic acid (SA) (García-Pérez et al., 2021b). For this, cells were vacuum filtered and transferred to 100-mL Erlenmeyer flasks at an initial cell density of 100 mg L^-1^, containing the same media supplemented with different concentrations of both elicitors, alone or in combination. MJ was tested at two concentrations, 50 and 100 µM. SA was tested at three concentrations, 100, 500, and 1000 µM. Stock solutions of MJ and SA were previously prepared in ethanol at 1 M and 0.1 M, respectively, filter-sterilized (0.22 µm pore size) and incorporated into the corresponding media. Cultures were incubated in the same conditions described above for eight days. After elicitation, cells were vacuum filtered, weighed (fresh biomass was expressed in grams of fresh weight, FW), and immediately frozen at -20°C. Frozen cells were lyophilized, powdered, and stored at -20°C until extraction. Elicitation experiments were carried out in triplicate.

### Sample extraction

Cell samples were subjected to solvent extraction using MeOH/HCOOH/H_2_O (80:0.1:19.9, v/v/v) at a concentration of 50 mg mL^-1^ in an ultrasonic bath at room temperature for 10 minutes. After centrifugation at 4 °C, 12,000 *g* for 10 minutes, the supernatants were syringe filtered (0.22 µm pore size) and stored in amber vials at -20°C until analysis.

### UHPLC-QTOF/MS phenolic profiling of elicited cell samples

The phenolic profile of cell extracts was obtained by an untargeted metabolomics approach through ultra-high performance liquid chromatography coupled to a quadrupole time-of-flight mass spectrometer (UHPLC-QTOF/MS). The chromatographic equipment consisted of a 1290 UHPLC system coupled to a JetStream electrospray ionization source and a G6550 QTOF spectrometer. First, a reverse phase chromatographic separation was carried out by applying a water-acetonitrile gradient, and compound detection was performed in SCAN mode (100-1200 m/z) at a nominal resolution of 40,000 FWHM (García-Pérez et al., 2021c). Moreover, quality controls were obtained by pooling an aliquot of each of the extracted samples, and analysed under the same chromatographic conditions, being acquired using data-dependent tandem mass spectrometry (MS^2^), as earlier explained in detail (Buffagni et al., 2022).

The annotation of phenolic compounds was achieved in accordance with Level-2 identification (putatively annotated compounds) according to the COSMOS Metabolomics Standard Initiative. For this purpose, annotation was performed by the Profinder (v. 10.0) software tool (Agilent), through mass (5-ppm tolerance) and retention time (0.05 min) alignment, using the database Phenol-Explorer 3.6, including the whole isotopic pattern of aligned features: monoisotopic accurate mass, isotopic pattern, and isotopic accurate spacing. Once annotated, the compounds were filtered by abundance, setting a signal-to-noise threshold of 8, and frequency, considering the features annotated in 75% of replicates within a treatment. After annotation, phenolic compounds were grouped into different subclasses and quantified according to calibration curves obtained with reference standards for each class. Tyrosol was selected as the standard for low molecular weight (LMW) phenolics, including alkylphenols, coumarins, phenylpropenes, quinones, tyrosols and other phenolics (y = 258779x), ferulic acid for phenolic acids (y = 677990x), sesamin for lignans (y = 229159x), *trans*-resveratrol for stilbenes (y = 387324x), luteolin for flavones, flavanones, isoflavonoids, and dihydrochalcones (y = 1000000x), quercetin for flavonols (y = 1000000x), cyanidin for anthocyanins (y = 3000000x), and catechin for flavanols (y = 415040x). Results were expressed as the equivalents of each reference standard, in mg per g of dry weight (DW), for instance: LMW content was expressed as tyrosols equivalents, TE; phenolic acid content was expressed as ferulic acid equivalents, FE; lignan content was expressed as sesamin equivalents, SE; stilbene content was expressed as *trans*-resveratrol equivalents, RE; flavone content was expressed as luteolin equivalents, LE; flavonol content was expressed as quercetin equivalents, QE; anthocyanin content was expressed as cyanidin equivalents, CyE; and flavanol content was expressed as catechin equivalents, CaE.

### Statistical analysis

Metabolomic profiling was achieved from raw data by applying the Agilent Mass Profiler Professional (v. 15.1) software tool. Filtered data were normalized at the 75^th^ percentile and the baseline was adjusted to the median of all samples. Afterward an analysis of variance (ANOVA, *α* = 0.05) followed by Tukey HSD *post-hoc* test was performed to identify the compounds with a statistically significant difference in terms of abundance for each treatment with respect to control, as well as a fold-change (FC) test (cut-off = logFC< 2) to identify the effect of each treatment on the abundance of every significant compound.

In addition, to decipher the different responses to elicitation treatments according to their metabolic fingerprint, a supervised orthogonal projection to latent structures discriminant analysis (OPLS-DA) was performed, using the SIMCA 16 software tool (Umetrics, Sweden). The resulting model was statistically validated by a cross-validation ANOVA (CV-ANOVA, *α* = 0.05), and the quality of fitness and predictability were assessed by the R^2^X, R^2^Y, and Q^2^Y parameters, respectively. Such modelling was combined with variable importance in projection (VIP) analysis committed to identifying the annotated compounds with the highest importance in the discrimination between elicitor treatments, selected according to their VIP score (threshold > 1.3).

The results for cell growth and phenolic compounds concentrations were analysed by a factorial ANOVA followed by Duncan’s *post hoc* test, using the SPSS (v. 25.0) software tool. The significance level was adjusted at *α* = 0.05.

### Modelling tools

After data acquisition, all experimental values were merged into a single database for modelling by NFL using the commercial software tool FormRules^®^ (v. 4.03). Three factors were designated as inputs (genotype, MJ, and SA), while nine parameters were selected as outputs (fresh biomass and the content of eight quantified phenolic subclasses). The adaptive-spline-modelling-of-data (ASMOD) algorithm was selected to ensure a minimal number of significant inputs to reduce model complexity and improve its accuracy with fewer parameters (Kavli and Weyer, 1995; Shao et al., 2006). The statistical fitness criterion selected was Cross Validation (CV) (Arlot and Celisse, 2010), which enabled the generation of robust models by avoiding data redundancy, since data are divided into several subsets (K parameter) to train the model and one subset that is randomly selected to test the trained model. Several values for the K parameter were tried to achieve quality models: K = 10 for FW and flavanols, K = 8 for flavones, and K = 9 for stilbenes. Other training parameters were: Adapt nodes: TRUE; Maximum inputs per submodel: 2; maximum nodes per input: 15.

The quality of submodels individually generated for each output was assessed, in terms of predictability and accuracy, by different parameters: The *f*-ratio is given by the ANOVA performed between the experimental and predicted values, the mean square error (MSE, Eq. 1), and the coefficient of determination (Train Set R^2^, Eq. 2).


$$MSE=\frac{\sum_{i=1}^{n}{\left(y_{i}-y_{i}\right)}^{2}}{n}$$

(Eq. 1)


$$R^{2}=\left(1-\frac{\sum_{i=1}^{n}{\left(y_{i}-y_{i}^{'}\right)}^{2}}{\sum_{i=1}^{n}{\left(y_{i}-y_{i}^{''}\right)}^{2}}\right)\times 100$$

(Eq. 2)

*y_i_* represents the experimental values, *y_i_’* represents the predicted values by the model, and *y_i_’’* represents the mean of the dependent variable.

Only robust models with high Train Set R^2^ values (>70%) were considered, due to their high accuracy and predictability. As well, models with Train Set R^2^ values >99.9% were rejected to avoid model over-fitting (Colbourn and Rowe, 2005; Landin et al., 2009). The model results were expressed by the generation of ‘IF-THEN’ rules, providing a defined interpretation of the predicted results, and combined with a certain range level (‘LOW’, ‘MID’, ‘HIGH’) and an individual membership degree, which ranges from 0 to 1 (Gallego et al., 2011). **Figure S1** includes the range of each output involved in the generation of the NFL model.

## Results and discussion

### Elicitation drives a genotype-dependent metabolic reprogramming for the accumulation of phenolic compounds in *Bryophyllum* PCSCs

The combination of untargeted metabolomics with neurofuzzy logic constitutes a promising cutting-edge approach to unravelling the phytochemical potential of little-explored medicinal plants. Their combination has recently been applied to decipher the effect of mineral nutrition on the biosynthesis of phenolic compounds from *Bryophyllum* medicinal plants cultured *in vitro* (García-Pérez et al., 2020c; García-Pérez et al., 2021c). Nevertheless, the exploitation of *Bryophyllum* as a source of natural bioactive compounds still requires further research. To that aim, in this work, a biotechnological system based on the elicitation of *Bryophyllum* PCSCs species was employed, using methyl jasmonate (MJ) and salicylic acid (SA) as enhancers of secondary metabolism.

**Table S1** includes the list of annotated compounds from the *Bryophyllum* cell extracts, where a total of 460 entities were determined among all treatments, together with their abundances, retention times, mass values, and molecular formulas. An ANOVA was performed for each species (*p<* 0.05; **Table S2**) to reveal those compounds with a statistically significant difference in terms of abundance due to elicitation with respect to control. Up to 77 significant compounds accounted for BD, 65 for BH, and only 22 for BT. These results indicate that the species BD and BH show a greater metabolic reprogramming than BT, which agree with previous results that showed a negligible effect of SA and MJ on the metabolome of BT cell cultures (García-Pérez et al., 2021b). Concerning the individual influence of elicitation treatments, **Table S2** shows the logFC values for the compounds significantly affected by every treatment with respect to control, to detect those concentrations and combinations responsible for the accumulation and/or inhibition of each compound biosynthesis.

For BD species, SA elicitation at 1000 µM led to the highest logFC values (37.6), specifically for flavonoids, such as kaempferol 7-*O*-glucoside, apigenin 7-*O*-glucuronide, and theaflavin (**Table S2**). Similarly, treatment with 100 µM MJ strongly induced the accumulation of quercetin 3-*O*-arabinoside (logFC = 38.9). In the combined treatments, mixtures of 500 µM SA and MJ at any concentration promoted phenolic compounds accumulation, as observed for peonidin 3-*O*-rutinoside for 50 µM MJ + 500 µM SA treatment (logFC = 35.6), and sesaminol and caffeic acid ethyl ester for 100 µM MJ + 500 µM SA treatment (logFC = 32.2 in both cases). The results suggest that the use of MJ or SA as single elicitors acts mainly on flavonoid biosynthesis, while their combination generates a more diversified metabolic response, involving phenolic acids, flavonoids, and lignans. The positive synergistic effect of MJ and SA combinations has been previously described for other *in vitro* systems, such as *Polyscias filicifolia* shoots, for which accumulation of phenolic acids occurred with the combination of 200 µM MJ and 50 µM SA (Śliwińska et al., 2021). For *Bryophyllum* species, the role of flavonoids in the stress response was previously reported for *in vitro*-grown plants of BD, for which flavonols, flavones, and anthocyanins biosynthesis was up-regulated under low nutrient conditions (García-Pérez et al., 2021a). Interestingly, the combination of MJ and SA followed an unclear pattern, highly dependent on the concentration of each elicitor. In fact, a strong inhibition of phenolic compounds biosynthesis, particularly from flavonoid subfamilies, was observed, especially in the treatments that combined the highest concentrations of elicitors (100 µM MJ + 1000 µM SA). This indicates that an excess of elicitors may contribute to the transition from *eustress* conditions to *distress* conditions, further leading to cell death *via* oxidative stress due to the inhibition of antioxidant compounds (Aguirre-Becerra et al., 2021).

For BH, 100 µM SA treatment caused a generalized up-regulation of phenolic compounds biosynthesis (logFC = 9.1), mostly represented by flavonoids, such as malvidin, quercetin, and derived glycosides, and lignans, represented by 5-pentacosenylresorcinol (logFC = 9.1; **Table S2**). Similarly, the highest logFC values (ranging from 13.3 – 24.9; **Table S2**) were observed in the combined treatment of 100 µM MJ and 500 µM SA, suggesting a synergistic effect of both elicitors, especially for secoisolariciresinol. This combination, however, strongly inhibits flavonoid biosynthesis (logFC = -6.0), indicating that phenolic compounds biosynthesis is strictly directed towards lignan production under elicitation and providing a promising natural source of these compounds with industrial purposes. PCSCs from several species under abiotic stress promote the elicitation of lignans, as it has been recently observed for SA-elicited *Linum album* PCSCs, *via* mitogen-activated protein kinase 6 (MAPK6) signalling (Samari et al., 2022).

Finally, the reduced number of compounds that are significantly affected by elicitation for BT indicates a slight metabolic reprogramming for this genotype. Myricetin biosynthesis is the only compound strongly inhibited by elicitation, with the lowest logFC values for 50 µM MJ + 500 µM SA treatment (-21.6). In contrast, the treatment with 1000 µM SA promoted the accumulation of the highest number of compounds, involving lignans (logFC ≈ 30), together with flavonoids and phenolic acids (logFC = 10.9 – 19.9), while the combination of 1000 µM SA with 50 µM MJ promoted the accumulation of the anthocyanin pelargonidin, and the flavanol epicatechin (logFC = 31.3 for both). The findings provide insights into the metabolomic response to elicitors, which was found to be multifaceted and genotype-dependent, even among closely related species, as shown for *Bryophyllum* in this study.

### Flavonoids, lignans, and stilbenes are revealed as metabolite markers of the elicitation-mediated reprogramming of *Bryophyllum* PCSCs

**Figure 1:**
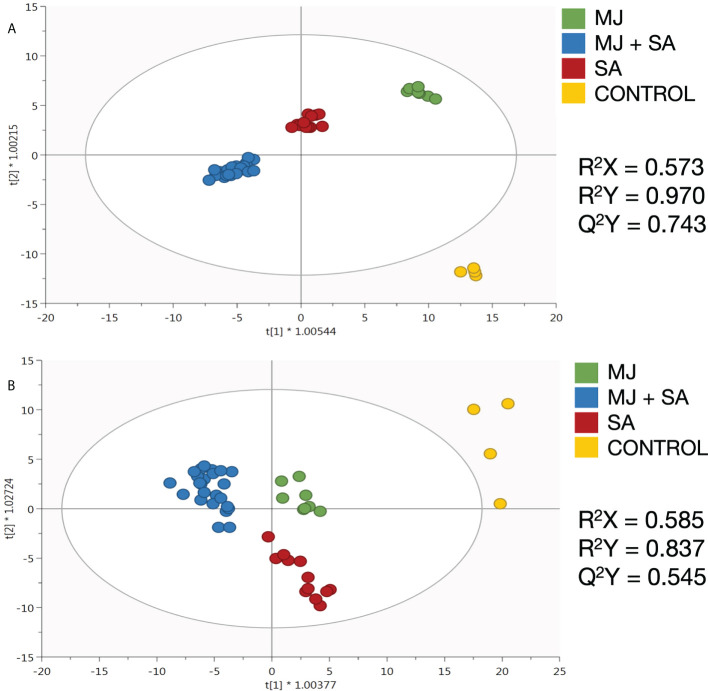
Orthogonal projection to latent structures models on the untargeted phenolic profile of elicited **(A)** *B. daigremontianum* and **(B)** *B.* x *houghtonii* cell suspension cultures, together with their quality parameters for goodness-of-fit (R^2^X and R^2^Y) and predictability (Q^2^Y). MJ, methyl jasmonate; SA, salicylic acid.

After deciphering the metabolic fingerprints of elicited *Bryophyllum* PCSCs, based on their phenolic composition, the development of supervised OPLS-DA models was performed to detect the compounds showing the highest contribution to the discrimination among elicitor treatments: control, MJ, SA, and MJ + SA. Moreover, a VIP analysis was further carried out to indicate the compounds with the highest influence in such discrimination, the so-called VIP markers. The OPLS-DA models presented high-quality values of goodness-of-fit (R^2^X and R^2^Y) and predictability (Q^2^Y) for BD and BH PCSCs (**Figures 1A, B**, respectively). In contrast, the model generated for BT was not robust (Q^2^Y< 0.5) and, therefore, was not considered due to its inability to discriminate between treatments. Consequently, the lack of such a predictive model did not make possible the determination of VIP markers for this species. This suggests that elicitors play a negligible role in the secondary metabolism of BT PCSCs.

The OPLS model generated for BD is shown in **Figure 1A**. The results indicate that elicitation played a definitive role in the metabolic fingerprint of these PCSCs, following the trend MJ< SA< MJ+SA, as these treatments were gradually separated from the control according to the principal latent vector (**Figure 1A**). These results agree with the previously reported evidence on the synergistic effect of MJ and SA on the secondary metabolism of BD (García-Pérez et al., 2021b). Additionally, discriminant VIP markers have been identified and shown in **Table 1**. Most of the metabolic markers found for BD are flavonoids, belonging to various subclasses, and other polyphenols, such as lignans and stilbenes (**Table 1**).

**Table 1:** Discriminant metabolites identified as VIP markers selected from the OPLS-DA model performed for the elicitation of BD PCSCs.

Compounds	Class	VIP Score^1^
Esculin	Hydroxycoumarins	1.60 ± 0.66
Rhoifolin	Flavones	1.54 ± 0.57
6’’-O-Malonylgenistin	Isoflavonoids	1.54 ± 0.66
Cinnamoyl glucose	Hydroxycinnamic acids	1.53 ± 0.63
Delphinidin 3-O-xyloside	Anthocyanins	1.50 ± 0.55
Phloridzin	Dihydrochalcones	1.50 ± 0.78
Naringenin 7-O-glucoside	Flavanones	1.47 ± 0.37
Dimethylmatairesinol	Lignans	1.43 ± 0.68
Dihydromyricetin 3-O-rhamnoside	Flavonols	1.43 ± 0.68
Demethyloleuropein	Tyrosols	1.41 ± 0.54
5-Heneicosenylresorcinol	Alkylphenols	1.39 ± 0.57
Hydroxytyrosol	Tyrosols	1.37 ± 0.39
Cyanidin 3-O-(6’’-acetyl-glucoside)	Anthocyanins	1.37 ± 1.36
Gallic acid 4-O-glucoside	Hydroxybenzoic acids	1.36 ± 1.12
1,4-Naphtoquinone	Naphtoquinones	1.36 ± 0.77
6-Geranylnaringenin	Flavanones	1.36 ± 0.77
p-Coumaroylquinic acid	Hydroxycinnamic acids	1.35 ± 0.91
Demethoxycurcumin	Curcuminoids	1.35 ± 0.90
Lariciresinol-sesquilignan	Lignans	1.34 ± 0.58
4-Vinylguaiacol	Alkylmethoxyphenols	1.33 ± 0.52
Pallidol	Stilbenes	1.33 ± 0.54
Ligstroside	Tyrosols	1.32 ± 1.23

^1^Each compound is accompanied by its corresponding VIP score ± standard error.

The OPLS model for BH also shows highly significant reprogramming after elicitation, although such a clear trend in discrimination versus control is not observed (**Figure 1B**). Polyphenols, mainly flavonoids, stilbenes, and lignans were identified from VIP markers as the most discriminating metabolites for BH PCSC (**Table 2**).

**Table 2:** Discriminant metabolites identified as VIP markers selected from the OPLS-DA model were performed for the elicitation of BH PCSCs.

Compounds	Class	VIP Score^1^
Cyanidin 3-*O*-sambubioside 5-*O*-glucoside	Anthocyanins	1.48 ± 1.17
Sesaminol	Lignans	1.48 ± 0.63
6-Geranylnaringenin	Flavanones	1.47 ± 0.69
Spinacetin 3-*O*-glucosyl-(1-6)-glucoside	Flavonols	1.47 ± 1.03
Ligstroside-aglycone	Tyrosols	1.46 ± 0.68
Resveratrol 3-*O*-glucoside	Stilbenes	1.43 ± 0.61
3-Caffeoylquinic acid	Hydroxycinnamic acids	1.42 ± 0.36
Valoneic acid dilactone	Hydroxybenzoic acids	1.41 ± 1.45
2,3-Dihydroxy-1-guaiacylpropanone	Hydroxybenzoketones	1.40 ± 0.46
Pallidol	Stilbenes	1.39 ± 0.53
Coumestrol	Other polyphenols	1.38 ± 0.63
Demethyloleuropein	Tyrosols	1.37 ± 1.47
Apigenin 7-*O*-glucoside	Flavones	1.35 ± 0.43
Isopimpinellin	Furanocoumarins	1.32 ± 0.63
Naringenin 7-*O*-glucoside	Flavanones	1.32 ± 0.49
(-)-Epicatechin	Flavanols	1.31 ± 0.66
Todolactol A	Lignans	1.30 ± 0.65

^1^Each compound is accompanied by their corresponding VIP score ± standard error.

Polyphenols were found as common markers on the elicitation of *Bryophyllum* PCSCs (**Tables 1** and **2**), for instance: cyanidin glucosides as anthocyanins, 6-geranylnaringenin and naringenin 7-*O*-glucoside as flavanones, and the stilbene pallidol, whereas ligstroside was a tyrosol also found as a discriminant for both species. Among them, flavonoids have been previously recognized as stress-derived metabolites in these *Bryophyllum* species, especially anthocyanins, flavones, and flavonols (García-Pérez et al., 2021a). However, to the best of our knowledge, flavanones have not yet been identified in the entire *Kalanchoe* genus. Such a response may eventually be due to the antioxidant activity of these compounds to counteract the oxidative burst driven by abiotic stress, as described in BD (Wang et al., 2018; García-Pérez and Gallego, 2022). In turn, lignans were recently found as responsible for the response towards sulfate deficiency in BH plants cultured *in vitro* (García-Pérez et al., 2021c) and they have been also found as leaf phytoconstituents of other *Kalanchoe* species, like *Kalanchoe gastonis-bonnieri* (Palumbo et al., 2019) and *Kalanchoe hybrida* (Kuo et al., 2018).

### The elicitation of *Bryophyllum* PCSCs is highly dependent on multiple factors and their interactions

**Figure 2:**
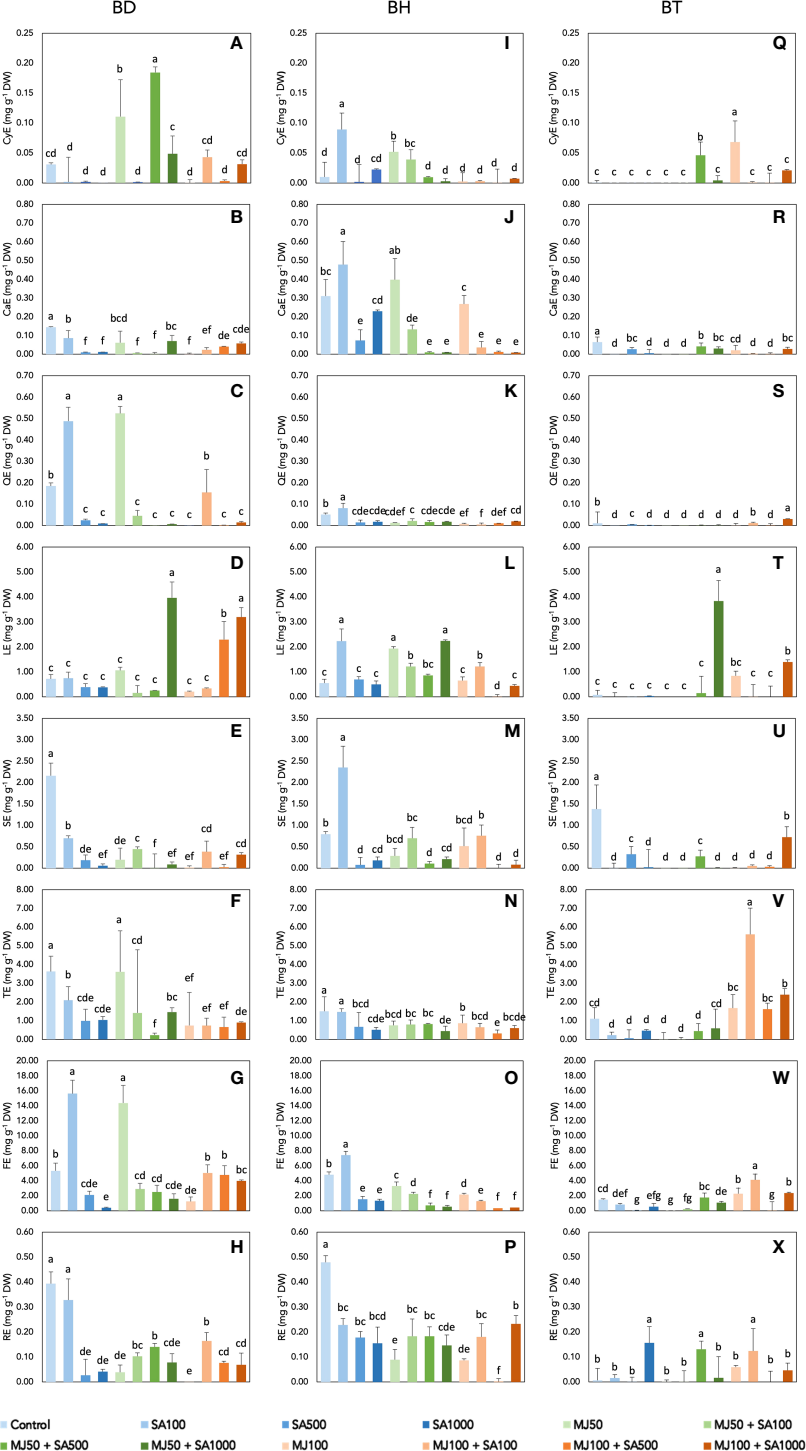
Semiquantification of phenolic compounds of elicited *Bryophyllum* PCSCs: **(A–H)** BD, **(I–P)** BH, and **(Q–X)** BT. The content of all phenolic subclasses was expressed in mg g^-1^ of dry weight (DW) as equivalents of a single representative standard: cyanidin equivalents (CyE) for anthocyanins; catechin equivalents (CaE) for flavanols; quercetin equivalents (QE) for flavonols; luteolin equivalents (LE) for flavones and related compounds; sesamin equivalents (SE) for lignans; tyrosol equivalents (TE) for LMW phenolics; ferulic acid equivalents (FE) for phenolic acids; and *trans-*resveratrol equivalents (RE) for stilbenes. Vertical bars indicate standard deviation. MJ, methyl jasmonate; SA, salicylic acid. Different letters indicate significant differences between elicitor treatments according to Duncan’s *post hoc* test (*p<* 0.05).

**Figure 2** shows the results for the semi-quantification of phenolic compounds by eliciting *Bryophyllum* PCSCs. To do this, the raw dataset including annotated compounds was filtered to remove isobaric compounds. Subsequently, the compounds were classified into 8 families: low-molecular-weight (LWM) phenolics, phenolic acids, lignans, stilbenes, flavones, and related compounds (flavanones, chalcones, and isoflavonoids), flavonols, anthocyanins, and flavanols. BD extracts showed the highest concentration of phenolics, in terms of phenolic acids, flavonols, and anthocyanins, while BH stood out as a rich flavanol, lignan, and stilbene source, and BT showed high contents of tyrosols and LMW phenolics (**Figure 2**). An ANOVA was performed to evaluate the significance of the involved factors (genotype, MJ, and SA) and/or their interactions with the accumulation of phenolic compounds (**Table S3**). The result of the analysis shows that all the variables, genotype, MJ, and SA, present a statistically significant effect on the concentration of all the phenolic subfamilies with *p*-values lower than 0.001 in almost all cases. The interactions between factors, i.e.: genotype × MJ, genotype × SA, MJ × SA, and genotype × MJ × SA also have a statistically significant effect (*p<* 0.001; **Table S3**). Such results support previous findings, in which different patterns of phenolic compound accumulation were observed for each *Bryophyllum* genotype studied (García-Pérez et al., 2020c).

Concerning the content of each phenolic subclass, anthocyanins were mostly found on BD-derived extracts, being elicited in the presence of 50 µM MJ in combination, and the maximum content was achieved by the combination of 50 µM MJ with 500 µM, 0.18 mg g^-1^ DW, showing a 5.9-fold increase with respect to the untreated control (**Figure 2A**). Flavanols were essentially found at high concentrations in BH extracts, being significantly accumulated under 100 µM SA and reaching the highest concentration, 0.48 mg g^-1^ DW (**Figure 2J**). Flavonols were reported at their highest content in BD extracts, and their content was significantly elicited by the lowest elicitor treatments, 100 µM SA and 50 µM MJ, accounting for up to ≅ 0.50 mg g^-1^ DW in both cases (**Figure 2C**). In the case of flavones, both BD and BT cells reached the highest contents after the elicitation with 50 µM MJ and 1000 µM SA: 3.97 mg g^-1^ DW (**Figure 2D**) and 3.84 mg g^-1^ DW (**Figure 2T**), respectively). With respect to lignans, only BH experience an elicitor effect, being positively affected by 100 µM SA, which allowed reporting the content of 2.35 mg g^-1^ DW (**Figure 2M**) whereas elicitation caused the inhibition of this phenolic subclass in the rest of the genotypes (**Figures 2E, U**). The highest content of LMW phenolics was found at BT extracts elicited with 100 µM MJ and 100 µM SA (**Figure 2V**), 5.62 mg g^-1^ DW, as a negligible effect was found for the rest of the genotypes (**Figures 2F, N**). Phenolic acids were significantly accumulated by elicitation, especially in BD cells at the mildest elicitor treatments, 100 µM SA and 50 µM MJ, reporting similar contents: 15.6 and 14.4 mg g^-1^ DW, respectively (**Figure 2G**). Finally, considering stilbenes, the accumulation of this subclass was essentially inhibited in the presence of elicitors, reporting its maximum in the control treatment of BH cultures, 0.48 mg g^-1^ DW (**Figure 2P**). Previous observations on *Bryophyllum* have indicated that its elicitation affects not only phenolic compounds, but also other stress-related compounds, such as glucosinolates, alkaloids, and triterpenoid phytoalexins (García-Pérez et al., 2021b). In this sense, the multifactorial effect of MJ and SA on the elicitation of medicinal plants has been also assessed through untargeted metabolomics approaches, such as in the case of *Bidens pilosa* (Ramabulana et al., 2020), supporting the great influence of the genotype on this phenomenon.

**Figure 3:**
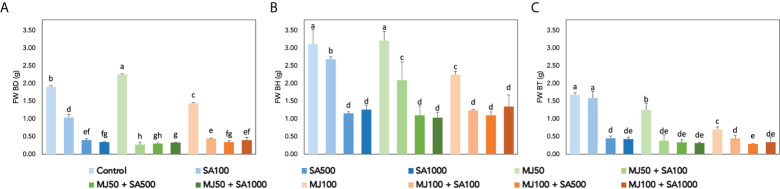
Fresh biomass determination of elicited *Bryophyllum* PCSCs, expressed as grams of fresh weight (FW) for **(A)** BD, **(B)** BH, and **(C)** BT. Vertical bars indicate standard deviation. Different letters indicate significant differences between elicitor treatments according to Duncan’s *post hoc* test (*p<* 0.05).

Elicitation affects the secondary metabolism but also other processes such as cell growth, as suggested in **Figure 3**, in which the fresh cell biomass was found to be affected after the different treatments are presented in a genotype and elicitor-dependent manner. The ANOVA results show that all the factors evaluated, as well as all their interactions, have a statistically significant effect on cell growth (*p*< 0.001; **Table S3**), suggesting a multifaceted elicitor behaviour. As a general trend, BH PCSCs showed the highest growth rates (>3.00 g), and it was maintained only after the treatment with 50 µM MJ, whereas the rest of the treatments led to a significant reduction in cell biomass (**Figure 3B**). In the case of BD PCSCs, 50 µM MJ caused a significant increase in cell biomass (**Figure 3A**), whereas BT cell biomass was also significantly reduced under elicitation with the exemption of 100 µM SA that exhibited sustained biomass with respect to control (**Figure 3C**). Accordingly, in all cases, combined treatments led to a strong inhibition potentially due to the transition from primary metabolism, involved in developmental functions, to secondary metabolism, mostly devoted to stress tolerance. The results are controversial when compared with previous studies in which cell growth proportional to nutrient consumption, in terms of dry weight, was observed in elicited *Bryophyllum* PCSCs. (García-Pérez et al., 2021b). Conversely, the elicitation of BH PCSCs with β-cyclodextrins promoted a significant increase in cell growth (García-Pérez et al., 2019).

The functional characterization of MJ and SA as elicitors of *Bryophyllum* PCSCs requires a detailed analysis of the influence of the independent factors as well as their concealed interactions, to get a valuable outcome with biological significance. To that aim, the application of machine learning technologies, such as NFL, constitutes a computer-based strategy to obtain deep knowledge and predict the role of elicitors on PCSCs growth and phenolic metabolism.

### Neurofuzzy logic provides a deep knowledge predicting the factors involved in the elicitation of *Bryophyllum* PCSCs

The results of cell growth and the semi-quantification of phenolic compounds of elicited *Bryophyllum* PCSCs, combined in a single database were modelled by NFL. Modelling results are shown in **Table 3**. Neurofuzzy logic provided a set of ‘IF-THEN’ rules that help to understand these effects in depth (**Table 4**).

**Table 3:** Critical factors and quality parameters detected by the NFL model for all the outputs evaluated.

Outputs	Submodel	Significant inputs	Train Set R^2^	MSE	*f* ratio	df1, df2	*f* critical(*α*=0.05)
FW	1	SA × Genotype	93.32	0.088	16.59	16, 19	2.22
	**2**	**MJ × SA**					
Anthocyanins	–	–	40.10	0.001	1.93	9, 26	2.27
Flavanols	**1**	**Genotype × SA**	78.71	0.005	5.55	14, 21	2.20
	2	MJ × Genotype					
Flavonols	–	–	54.86	0.010	2.33	12, 23	2.20
Flavones	1	SA × Genotype	77.18	0.440	5.07	14, 21	2.20
	**2**	**MJ × SA**					
Lignans	–	–	63.36	0.220	1.83	17, 18	2.23
LMW	–	–	59.68	0.000	0.00	35, 0	0.00
Phenolic acids	–	–	48.27	0.006	2.04	11, 24	2.21
Stilbenes	**1**	**MJ × SA × Genotype**	88.74	0.006	2.34	27, 8	3.10

df, degrees of freedom; FW, fresh weight; LMW, low molecular-weight compounds; MSE, mean square error. Bold inputs indicate the strongest effect associated with each output

**Table 4:** Complete set of “IF-THEN” rules obtained by NFL models with their corresponding degrees of membership (MD).

Rules		Genotype	SA	MJ		FW	Flavanols	Flavones	Stilbenes	MD
1	IF	BD	LOW		THEN	LOW				0.84
2		BH	LOW			HIGH				0.99
3		BT	LOW			LOW				1.00
4		BD	MID			LOW				1.00
5		BH	MID			LOW				0.65
6		BT	MID			LOW				1.00
7		BD	HIGH			LOW				1.00
8		BH	HIGH			LOW				0.51
9		BT	HIGH			LOW				1.00
**10**		** **	**LOW**	**LOW**		**HIGH**				**1.00**
11			MID	LOW		LOW				0.95
12			MID	LOW		LOW				0.74
13			HIGH	LOW		LOW				0.85
14			LOW	HIGH		HIGH				0.53
**15**		** **	**MID**	**HIGH**		**LOW**				**1.00**
16			MID	HIGH		LOW				0.80
17			HIGH	HIGH		LOW				0.84
18	IF	BD	LOW		THEN		LOW			0.85
19		BD	MID				LOW			1.00
20		BD	HIGH				LOW			0.91
**21**		**BH**	**LOW**	** **			**HIGH**	** **	** **	**0.92**
**22**		**BH**	**MID**	** **			**LOW**	** **	** **	**1.00**
23		BH	HIGH				LOW			1.00
24		BT	LOW				LOW			0.98
25		BT	MID				LOW			0.97
26		BT	HIGH				LOW			0.96
27		BD		LOW			LOW			0.83
28		BH		LOW			HIGH			0.74
29		BT		LOW			LOW			0.93
30		BD		HIGH			LOW			0.97
31		BH		HIGH			LOW			1.00
32		BT		HIGH			LOW			0.98
33	IF	BD	LOW		THEN			LOW		0.90
34		BH	LOW					HIGH		0.51
35		BT	LOW					LOW		1.00
36		BD	HIGH					HIGH		0.83
37		BH	HIGH					LOW		0.90
38		BT	HIGH					LOW		0.61
39			LOW	LOW				LOW		0.80
40			MID	LOW				LOW		1.00
**41**		** **	**HIGH**	**LOW**				**LOW**	** **	**1.00**
42			LOW	MID				LOW		0.76
43			MID	MID				LOW		1.00
**44**		** **	**HIGH**	**MID**				**HIGH**	** **	**1.00**
45			LOW	HIGH				LOW		
46			MID	HIGH				LOW		
47			HIGH	HIGH				LOW		
**48**	IF	**BD**	**LOW**	**LOW**	THEN				**HIGH**	**0.83**
49		BH	LOW	LOW					HIGH	0.81
50		BT	LOW	LOW					LOW	0.98
51		BD	MID	LOW					LOW	0.94
52		BH	MID	LOW					LOW	0.68
53		BT	MID	LOW					LOW	1.00
54		BD	HIGH	LOW					LOW	0.91
55		BH	HIGH	LOW					LOW	0.68
56		BT	HIGH	LOW					LOW	0.67
57		BD	LOW	MID					LOW	0.88
58		BH	LOW	MID					LOW	0.74
**59**		**BT**	**LOW**	**MID**					**LOW**	**1.00**
60		BD	MID	MID					LOW	0.69
61		BH	MID	MID					LOW	0.60
62		BT	MID	MID					LOW	0.73
63		BD	HIGH	MID					LOW	0.84
64		BH	HIGH	MID					LOW	0.69
65		BT	HIGH	MID					LOW	0.96
66		BD	LOW	HIGH					LOW	0.85
67		BH	LOW	HIGH					LOW	0.71
68		BT	LOW	HIGH					LOW	0.80
69		BD	MID	HIGH					LOW	0.80
70		BH	MID	HIGH					LOW	0.97
71		BT	MID	HIGH					LOW	0.98
72		BD	HIGH	HIGH					LOW	0.86
73		BH	HIGH	HIGH					LOW	0.51
74		BT	HIGH	HIGH					LOW	0.90

The rules indicated in bold correspond to the submodels with the greatest positive or negative effect on each output. SA, salicylic acid; MJ, methyl jasmonate; FW, fresh weight.

Four out of nine outputs were efficiently modelled with high predictability and accuracy. Train Set R^2^ values higher than 70% were found for cell growth expressed as fresh weight, and the content of three phenolic subclasses, including flavanols, flavones, and related compounds (flavanones and isoflavonoids), and stilbenes (**Table 3**). Fortunately, the model was able to predict the content of three polyphenolic subclasses, which were previously identified as metabolic markers involved in the response to elicitation (**Tables 1** and **2**). However, other phenolic families were not efficiently predicted (Train Set R^2^< 70%), which can be due to the influence of other factors on their biosynthesis, such as the heterogeneity of compounds classified as LMW compounds, or the composition of culture media that has been also proven to have a definitive effect on the phenolic metabolism of *Bryophyllum* plants cultured *in vitro* (García-Pérez et al., 2021c).

SA was spotted as a critical factor for all the efficiently predicted outputs, suggesting its importance on cell growth and phenolic biosynthesis of *Bryophyllum* PCSCs, always conditioned by genotype. The combination of SA with MJ also plays a definitive role in cell growth, flavones, and stilbenes content (**Table 3**). Consequently, the generated model indicates that SA promotes a fine-tuning metabolic reprogramming of elicited *Bryophyllum* PCSCs.

Variations in cell growth (FW) are explained in a 93.32% mainly as a function of the interaction between both elicitors and also the interaction of genotype and SA (**Table 3**). For the cell growth model (FW) the rules fully agree with the conclusions derived from **Figure 3**, being the combination of LOW levels of elicitors the ones that produce the highest cell growth rates (rule 10, **Table 4**). The rules also corroborate the higher cell growth for the BH genotype (rule 2, **Table 4**) and suggest that SA is the major responsible for the inhibition of cell growth since mid and high of this elicitor always promoted LOW growth rate (rules 11-13, 15-17; **Table 4**), which correspond to the LOW SA concentration (range of 125 – 1000 µM; **Figure S1**), with the independence of MJ concentrations. These results are in accordance with previous evidence, supporting that MJ is recognized as a central regulator of secondary metabolism, thus leading to the shift from primary metabolism to secondary metabolism involved in the modulation of stress-related responses. In this sense, several authors have displayed contrasting results, reporting a negative impact of MJ on medicinal PCSCs growth: 100 µM MJ inhibited *Gardenia jasminoides* cell growth (Liu et al., 2018), meanwhile 50 µM MJ inhibited *Buddleja cordata* cell culture growth (Arano-Varela et al., 2020).

In agreement with the results presented in **Figure 3** for flavanols biosynthesis, the NFL model for this parameter points out the major role of the interaction genotype × SA in explaining these subclass variations, while the interaction MJ × genotype was predicted as a secondary factor (**Table 3**). Little is known about the production of flavanols by *Bryophyllum* sp., since it has been recently reported for the first time in the leaves of BD and roots of BT cultured *in vitro* (García-Pérez et al., 2021c). NFL results support these previous findings about the genotype-dependent biosynthesis of flavanols by these species. Accordingly, (-)-epigallocatechin was found to be accumulated in 50 µM MJ-elicited BH PCSCs, which is the major species revealed by the model to accumulate HIGH flavanol concentrations under LOW SA concentration (<250 µM, **Figure S1**; rule 21 **Table 4**). The results reported here indicate an inhibitory effect of SA at HIGH concentrations, which agrees with Gadzovska et al. (2013) who proved that SA is not a universal elicitor, so it can exclusively stimulate certain subfamilies of secondary metabolites. Consequently, the choice of elicitors for modulation of phenolic compounds biosynthesis by PCSCs should be thoroughly optimized for each application.

Flavones model provided two submodels, being the interaction MJ × SA the most relevant effect, together with the interaction genotype × SA to a lesser extent (**Table 3**). Interestingly, the rules defined for this subfamily indicate a fine-tuning modulation of their biosynthesis guided by elicitation, since only HIGH flavone concentrations are found for the combination of HIGH SA concentrations (>750 µM) and MID MJ concentrations (25 – 75 µM, rule 44; **Table 4**). The application of NFL provides useful information on the operational conditions required to maximize the biosynthesis of this subfamily, which includes, in addition to flavones, other related compounds (isoflavonoids, flavanones, etc.). The combined use of MJ and SA as elicitors of *Bryophyllum* PCSCs significantly promotes flavone and isoflavone biosynthesis, led by a differential contribution of each elicitor (García-Pérez et al., 2021b). The conditions indicated by the model to obtain high flavone concentrations are in agreement with previous results obtained for other *in vitro* systems, such as hairy roots of *Astragalus membranaceus* (Gai et al., 2016), and *Pueraria candollei* (Udomsuk et al., 2011).

Finally, the interaction of the three factors involved in this work (MJ × SA × Genotype) accurately predicts (Train Set R^2^ = 88.74%) the stilbene biosynthesis (**Table 3**). The rules for this output indicate that BD and BH genotypes lead HIGH stilbenes concentrations when LOW MJ and SA concentrations were used for elicitation (rules 48 and 49, respectively, **Table 4**). However, BT PCSCs always display LOW values regardless of the treatment applied (**Table 4**). It has been reported that stilbene biosynthesis by PCSCs, is more efficient when elicitors are combined with other stimuli. Examples of this are the combination of elicitors with ultraviolet C light in *Vitis vinifera* PCSCs (Xu et al., 2015), or that of sucrose and biotic elicitors in *Cayratia trifolia* PCSCs (Arora et al., 2010). On this basis, the optimization of stilbenes by *Bryophyllum* PCSCs should involve not only a deep characterization of interactions between all factors implicated in the stress-driven response to elicitors but also the evaluation of elicitors from different origins and mechanisms of action.

## Conclusions

The combination of plant tissue culture, metabolomics, and machine learning have been revealed as a robust methodology to study the potential to produce phenolic compounds and/or their improvement by elicitation on the PCSCs of *Bryophyllum* sp. This process presents a multifactorial behaviour driven by complex interactions between all the factors involved (genotype, SA, and MJ). The application of UM provided insights into the metabolic fingerprinting associated with elicitation, focused on the biosynthesis of phenolic compounds by *Bryophyllum* sp., including flavanones that were identified for the first time in the entire *Kalanchoe* genus.

The NFL technique has proved useful to predict and further characterize elicitation in *Bryophyllum* PCSCs, conferring a deep knowledge of this process even with a relatively simple experimental design. The use of MJ and SA as elicitors promotes a genotype-dependent reprogramming that impacts cell growth and phenolic metabolism. SA plays an important role as an elicitor of phenolic compound biosynthesis, following a fine-tuning mechanism of action, while MJ was predicted as a secondary factor. The production of flavones, isoflavones, flavanones, stilbenes, and flavanols was efficiently predicted by this multifaceted strategy. This represents an enormous potential for the large-scale exploitation of *Bryophyllum* PCSCs as biofactories to efficiently produce compounds under elicitation to be applied in the food, nutritional, and/or cosmetic industries.

## Data availability statement

The original contributions presented in the study are included in the article/**Supplementary Material**. Further inquiries can be directed to the corresponding authors.

## Author contributions

Conceptualization, PG-P, LL, and PG. Methodology, PG-P, EL-M, LZ, BM-M, and ML. Software, PG-P, LZ, BM-M, ML, and LL. Formal analysis, PG-P, EL-M, and LZ. Investigation, PG-P, EL-M, and PG. Resources, LL and PG. Data curation, PG-P, ML, and LL. Writing-original draft preparation, PG-P and EL-M. Writing-review and editing, ML, LL, and PG. Supervision, LL and PG. Project administration, LL, and PG. Funding acquisition, LL, and PG. All authors contributed to the article and approved the submitted version.

## Funding

This work was funded by Xunta de Galicia through the Cluster of Agricultural Research and Development (CITACA Strategic Partnership, grant number ED431E 2018/07) and “Red de Uso Sostenible de los Recursos Naturales y Agroalimentarios” (REDUSO, grant number ED431D2017/18).

## Acknowledgments

The authors acknowledge the “Margarita Salas” grant awarded to PG-P, supported through the European Union by the “NextGenerationEU” program.

## Conflict of interest

The authors declare that the research was conducted in the absence of any commercial or financial relationships that could be construed as a potential conflict of interest.

## Publisher’s note

All claims expressed in this article are solely those of the authors and do not necessarily represent those of their affiliated organizations, or those of the publisher, the editors and the reviewers. Any product that may be evaluated in this article, or claim that may be made by its manufacturer, is not guaranteed or endorsed by the publisher.
