# Supplementary material for: Neurofuzzy logic predicts a fine-tuning metabolic reprogramming on elicited Bryophyllum PCSCs guided by salicylic acid
Source: Front Plant Sci. 2022 Sep 23;13:991557. doi: 10.3389/fpls.2022.991557 (PMC9541431; doi:10.3389/fpls.2022.991557)
Supplement: Supplementary file 1 [file DataSheet_1.docx]

Supplementary Material

# Supplementary Table S1. Full dataset on the chemical entities provided by UHPLC-QTOF/MS untargeted metabolomics approach. Compound names are given their abundances (in total ion current, TIC) for each treatment (n = 4 replicates), as well as their retention time (min), mass (u), and molecular formula.

**Supplementary Table S2.** Statistically significant compounds given by the ANOVA (*α* = 0.05) analysis for the comparison between elicitor treatments and untreated controls on *Bryophyllum* PCSCs. For each compound, *p*-value was calculated, as well as their normalized logFC values with respect to control.

**Supplementary Table S3.** Statistical parameters provided by the ANOVA performed on cell growth and the content of different phenolic subclasses of elicited *Bryophyllum* PCSCs. Gen.: genotype; MJ: methyl jasmonate; SA: salicylic acid; SS: sum of squares; df: degrees of freedom; MS: mean square; F: statistical F value; p: probability.


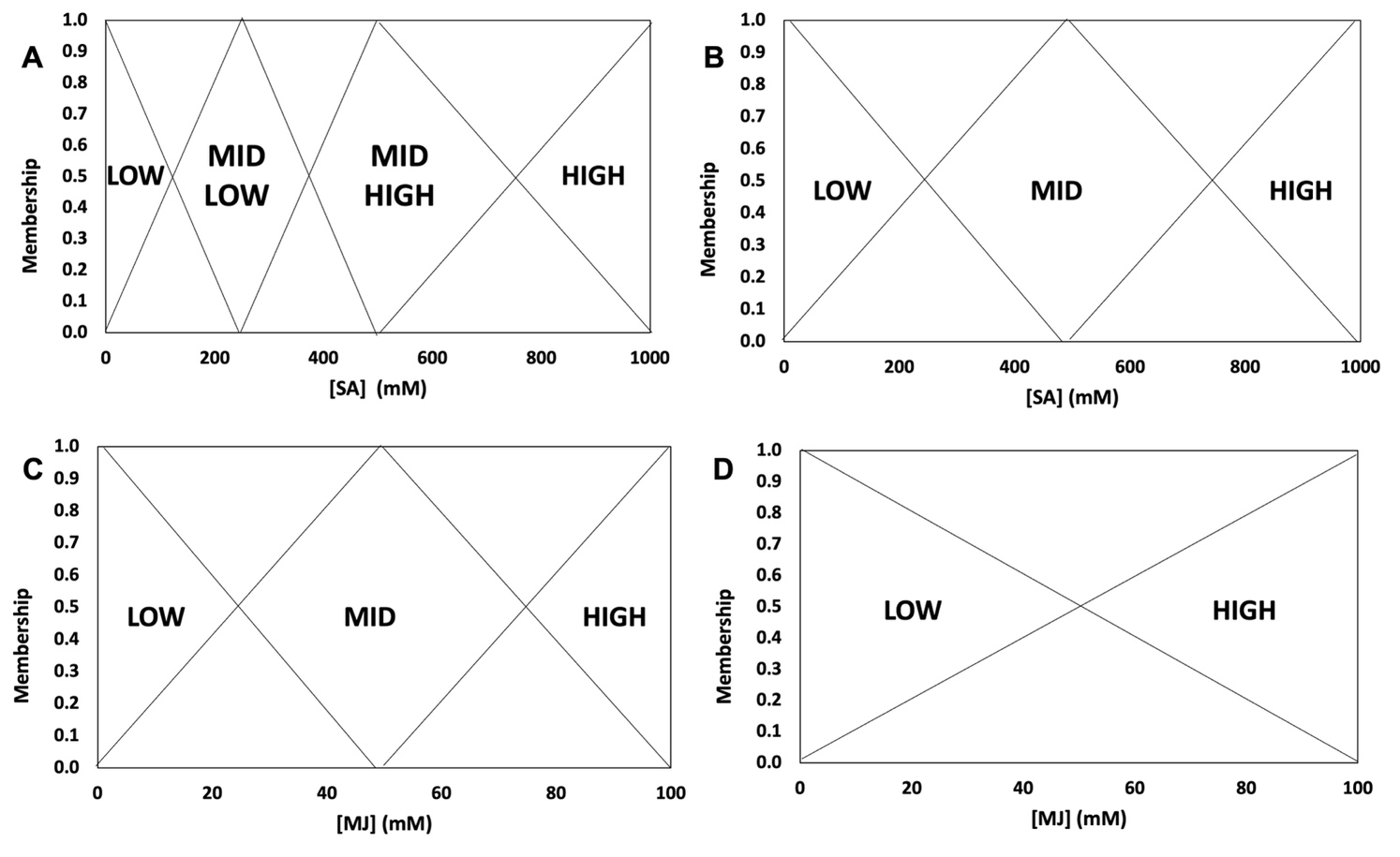


**Supplementary Figure S1.** Graphical ranging of each elicitor given by ANN model for every output: **(A)** SA concentrations for FW; **(B)** SA concentrations for flavanol, flavone, and stilbene contents; **(C)** MJ concentrations for flavone and stilbene contents; **(D)** MJ concentrations for FW.
